# Associative Learning in Subclinical Hypothyroidism at Different Ranges of Thyroid Stimulating Hormone: A Cambridge Neuropsychological Test Automated Battery (CANTAB) Study of Visual Paired Association Learning Task

**DOI:** 10.2174/0118715303312982240902102722

**Published:** 2024-09-23

**Authors:** Satkarjit Kaur Jhandi, Shweta Shenoy, Ajaypal Singh

**Affiliations:** 1 MYAS-GNDU Department of Sports Sciences and Medicine, Guru Nanak Dev University, Amritsar, Punjab, 143005, India;; 2 Outpatient Department and Emergency Branch, Apollo Hospital Amritsar, Punjab, 143005, India

**Keywords:** Associative learning, memory retrieval, subclinical hypothyroidism, Alzheimer’s disease, CANTAB, euthyroid, levothyroxine

## Abstract

**Background:**

Associative learning deficits are constantly found in subclinical hypothyroidism (SCH). Despite achieving normal thyroid stimulating hormone (TSH) levels, a considerable number of patients undergoing levothyroxine (LT-4) treatment frequently complain about memory retrieval. The Paired Association Learning (PAL) task involves computerised testing on the CANTAB- Cambridge Neuropsychological Test Automated Battery, also considered a screening tool for Alzheimer’s disease (AD).

**Purpose:**

This study aimed to investigate the impact of different levels of TSH on visual associative learning in SCH and determine if these impairments were reversed with LT-4.

**Methods:**

A total of 134 participants were included in this cross-sectional study. Group 1: 35 healthy controls; patients with SCH (Group 2: 33 newly identified cases; Group 3: 32 patients on LT-4 with elevated TSH; Group 4: 34 euthyroid but on LT-4). A thyroid profile and a neuropsychological clinical assessment were done. The visual PAL task was performed on CANTAB.

**Results:**

PAL was significantly impaired (*p* = <0.05) in all 3 patient groups as compared to Group 1. The PAL total errors (adjusted) scores were significantly higher in Groups 2 and 3, indicating that associative learning is definitely impaired in SCH, reaching levels previously seen in patients with AD.

**Conclusion:**

Our findings encourage screening for visual associative learning or memory retrieval in patients with SCH. The study present has established a more reasonable threshold of TSH 2.5mIU/L to encourage examination of associative learning and the initiation of LT-4 in SCH. Poor PAL task performance in patients with SCH may have significant implications in clinical settings for suspecting AD.

## 1 INTRODUCTION

Hypothyroidism is often accompanied by neurocognitive and neuropsychiatric symptoms, indicating the crucial role of thyroid hormones in maintaining healthy brain function [1]. This condition can impact general intelligence, psychomotor speed, visual-spatial skills, and memory [2].

Some studies [3-6] concluded that patients with hypothyroidism have impairment in various cognitive domains, such as executive functions, working memory, attention, and learning abilities. An imaging study [7] demonstrated that alterations in intrinsic resting-state functional connectivity within the somato-motor and right frontoparietal networks in patients with subclinical hypothyroidism (SCH), may contribute to the decline of cognitive abilities observed in these patients. Some other cross-sectional studies [8, 9] also concluded that there was a significant association between cognitive impairments and SCH.

Hypothyroid-related memory deficits are attributed to a specific deficit in memory retrieval or paired associative learning (PAL) [2]. PAL is a traditional memory model that explains how humans encode and retrieve recently formed associations between stimuli. It has been widely employed to investigate and comprehend the mechanisms of information learning and forgetting. Because traditional unrelated concepts are used in paired-associate learning paradigms. PAL has been considered for items such as unrelated words or number-letter combinations as a technique for investigating the mechanisms that underpin human memory without the confusing forces of earlier learning history that occur when pairs of concepts have a prior association [10].

The PAL task of the Cambridge Neuropsychological Test Automated Battery (CANTAB) is essentially a visual pair-association learning task that measures conditional learning of pattern-place associations. It is assumed that it specifically examines medial temporal, *i.e*., hippocampal, function. Memorising items in space is a well-known activity that activates the hippocampus. Particularly, the types of learning associated with reading acquisition have been investigated through the use of PAL tasks. Learning and recalling the connections between artificially connected stimuli-such as abstract figures and pseudowords-are key components of the PAL tasks.

The PAL task has been shown to be able to distinguish between healthy individuals and patients with Alzheimer’s disease (AD) with high sensitivity and specificity. A PAL total (adjusted) error of 45.5 had a sensitivity of 96% and a specificity of 77%, while a score of 72 had a sensitivity of 80% and a specificity of 96%, suggesting that the PAL task could be used as a stand-alone screening test for AD [11]. Another study [12] reports that a cut-off score of 71 PAL total errors (adjusted) distinguishes between healthy adults and individuals with AD and mild cognitive impairment (MCI).

From a clinician’s perspective, SCH is of great interest because of its high prevalence [13] and its high association with neuropsychological symptoms (such as fatigue, insomnia, depressive mood, apathy, and mental fogginess) [14, 15], as well as cognitive impairments [16-19].

The impairment of associative learning in hypothyroidism has been repeatedly investigated in the past. A study [20] reported that there was a correlation between hypothyroidism and significant impairment in verbal associative learning and retention, which improved with treatment. Another study [21] reported a significant improvement in verbal memory using a memory composite of contextual and associative memory tasks. Few authors [22] found that discontinuation of thyroid hormone treatment in patients with thyroid cancer was associated with impaired performance for specific verbal memory deficits memorised in a list learning task, whereas no significant memory deficits occurred with successful treatment. In contrast to those reporting improvement with treatment, a study by Leentjens & Kappers [23] reported that in specific case studies, basic memory functions remained impaired after treatment compared to healthy individuals. Furthermore, several recent studies [24-26] (Allam *et al*., 2008; Goyal *et al*., 2018; Parle *et al*., 2010) have identified that individuals with hypothyroidism experience challenges in associative learning and memory retrieval.

Improvements in cognitive functions in patients who are having levothyroxine (LT-4) treatment are frequently overlooked since many patients continue to exhibit cognitive dysfunction even after the TSHs level returns to normal *i.e*., euthyroid state [18, 27], indicating that the abnormal TSHs level may have long-term consequences on cognitive functions. Talaie *et al*. [28] have documented that individuals with a TSH ≤ 4.0 mIU/L and no symptoms should be considered euthyroid. However, if an individual reports neuropsychological symptoms, a cut off value of TSH 2.5mIU/L should be used as a guideline for starting LT-4 treatment. Another study by Hayashi *et al*. [29] reported that a range of TSH 0.45-4.49 mIU/L should be considered as euthyroidism (with no symptoms), while TSH 4.5-19.9 mIU/L was categorized as SCH, with free thyroxine (fT4) values within the reference range (5.0 to 12.0 μg/dL). Considering this background in mind, patients should be evaluated at these TSH ranges to see whether learning deficits occur in patients with SCH.

The current study aimed to investigate the impairment of visual, associative learning in SCH at different TSH levels and whether these deficits are reversed in patients having LT-4. The outcomes of the study carry implications for clinical practice, particularly in terms of the prognosis of hypothyroidism and the possible long-term cognitive problems that patients may experience.

Though designed as a cross-sectional study, the time duration patients were on LT-4 treatment was also considered to examine the effects on visual paired associative learning. The automated version of CANTAB PAL was used in four different groups to look for an association between TSH levels and performance of PAL, as well as enhancement of PAL performance scores associated with the duration of treatment.

Therefore, the current study explored: the visual paired associative learning in patients with elevated TSH (≥4.0mIU/L) and euthyroid (TSH, <4.0 mIU/L) compared to newly identified cases (TSH, ≥2.5 mIU/L) and healthy controls.

## 2 METHODS

### 2.1 Participants and Study Design

The study design is cross-sectional; it involved 99 diagnosed patients. They were recruited from several health centers in Amritsar and were also recruited through local general practices in the period of May 2022 to July 2023. Formal written authorization was obtained from each consultant physician to initiate the recruitment process.

Thirty-five healthy individuals matching age, gender and educational level were recruited from the local community and considered as a control group. These individuals exhibited normal results in thyroid function tests and possessed a medical history characterized by good overall health, absence of neurological and significant cardiovascular disorders, and lack of uncontrolled chronic conditions.

All participants gave their informed consent before participating in the study. This study was carried out in accordance with the Declaration of Helsinki and approved by the Ethics Committee of Guru Nanak Dev University, Amritsar (CTRI/2022/04/042319). The sample size estimation was done with the help of G* Power (3.1.9.2), wherein ρ= 0.30 (4); d = 0.05 (absolute error) and β = 0.84 were taken and estimated as 134 of the total sample. The aim was to study more than 30 patients in each group and as healthy controls.

### 2.2 Subjective Complaints and Biochemical Measures

Self-developed assessment form, including all the hypothyroidism and neuropsychological symptoms, was given to the patients and they were asked about their subjective complaints, with a focus on complaints that had arisen or been exacerbated as a result of thyroid pathology. Each patient had their thyroid functions clinically and biochemically evaluated. The estimation of serum levels of TSH in mIU/L and free T3 (fT3) in ng/mL, free T4 (fT4) in µg/dL of all the participants were done with mini VIDAS (automated immunoassay system) by the certified medical technologist. The blood samples were collected in the morning, around 8:00 am to 10:00 am, with fasting status of the patient.

Patients' medical comorbidities, personal history for psychiatric illnesses, familiarity with psychiatric disorders, and concurrent intake of therapy (psychiatric and nonpsychiatric medicines) were all noted. The medical history of the patients revealed the presence of a continuing LT-4 treatment regimen, provided at particular doses, as well as the duration of the treatment, history of thyroid replacement, and neuropsychological symptoms.

### 2.3 Clinical Evaluation and Recruitment

Patients with SCH were diagnosed based on thyroid function test results and their reported symptoms or complaints.

The patients who came up with primary complaints of neuropsychological symptoms (such as fatigue, forgetfulness, insomnia, and depressive symptoms) and with TSH ≥2.5 mIU/L, the consultants identified them as newly diagnosed cases with SCH.

The patients with hypothyroidism symptoms (such as fatigue, dry skin, cold intolerance, weight gain) or uncontrolled TSH levels (≥4.0mIU/L) were enrolled as patients with elevated TSH levels. The patients recruited as euthyroid with the range of < 4.0mIU/L TSH level were seen for follow-up.

### 2.4 Eligibility Criteria and Classification of Groups

Participants were included and grouped on the basis of their symptoms, TSH values and medication history. Mini Mental State Examination (MMSE) scored more than 24 to all the participants and they were made to abstain from the consumption of caffeine and nicotine for at least 12 hours before CANTAB procedure.

Group 1: Controls- Healthy Individuals (with no history of thyroid dysfunction).

Group 2: newly identified cases with TSH levels ≥ 2.5 mIU/L presenting with neuropsychological symptoms such as depression, insomnia, irritability, feelings of guilt, anxiety, forgetfulness but with no past history of thyroid dysfunction).

Group 3: Patients with presenting complaints of hypothyroidism symptoms (such as weight gain, fatigue, cold intolerance, dry skin, constipation, lack of memory) and TSH levels ≥4.0mIU/L (ongoing LT-4 treatment because of poorly controlled TSH levels).

Group 4: Euthyroid patients with TSH levels <4.0mIU/L and symptoms of lack of memory, insomnia, depressive mood, currently under LT-4 treatment to maintain TSH levels within normal range.

Exclusion Criteria: Patients with any other neurological disorders or any other psychiatric disorders; drug history of antipsychiatry; presented with a family history of dementia; any other systemic diseases such as myocardial infarction, hypertension or diabetes mellitus; any drug addiction.

### 2.5 Objective Cognitive Measures, Socioeconomic Status, Level of Education

Cognitive status was assessed using the Mini Mental State Examination (MMSE), thirty is the maximum score. It consists of 3 categories for cognitive impairment severity: 0-17 represents that the cognitive impairment is severe, 18-23 represents cognitive impairment is mild, and 24-30 represents there is no cognitive impairment [30]. Participants could enroll if they scored more than 24.

Socioeconomic status was assessed with the Kuppuswamy scale [31], the total score ranges from 3 to 29. It categorizes families into 5 different groups, “upper class (I), upper middle class (II), lower middle class (III), upper lower (IV) and lower socio-economic class (V) on the basis of a) occupation of the family head, b) education of the head of the family, c) monthly income of the family.”

Education level was recorded for each participant (level 1, left formal education before age 16; level 2, left formal education at age 16; level 3, left formal education at age 17-18; level 4, undergraduate degree or equivalent; level 5, Master’s degree or equivalent; level 6, PhD or equivalent).

### 2.6 Examination of Visual Paired Associative Learning

All the participants were administered the computerized neuropsychological test for paired associative learning. CANTAB was administered for PAL task to the study participants.

An acquisition phase followed with a recall phase was used in this task. The patterns were displayed in box sequence during acquisition phases 2, 4, 6, or 8. This was followed by the recall phase, in which the subject had to accurately identify the starting position of the patterns shown. If the participant successfully identified the positions of the patterns, the test proceeded to the next stage; if an error was made, the acquisition and recall stages were repeated. Each level allowed 10 trials before the test was automatically terminated and a total score was calculated. The following outcomes were recorded:

1. PALTE-PAL Total Errors PALTE: The total number of times a respondent selected an incorrect box when trying to remember the location of a pattern. Calculated overall scored trials.
2. PALTEA- PAL Total Errors (Adjusted): The number of times the subject selected the wrong box for a stimulus on the assessment tasks (PALTE) plus an adjustment for the estimated number of errors they would have made on all tasks, trials and recalls that they did not achieve. In this way, the error performance of all subjects was measured, regardless of whether they completed the task early or played through to the end.
3. PALTA-PAL Total Attempts: The total number of tries the individual made (but did not necessarily complete) during the assessment tasks.
4. PALFAMS-PAL First Attempt Memory Score: The number of times a subject selected the correct box on the first trial when remembering the positions of the patterns. Calculated overall scored trials.
5. PALMETS-PAL Mean Errors to Success: The average number of attempts a subject needs to successfully complete the stage.

### 2.7 Statistical Analysis

SPSS version 27.0 (IBM SPSS Statistics for Windows, [IBM Corp., Armonk, NY. USA]) was used for the statistical analysis. Descriptive statistics were employed to elucidate the fundamental characteristics of the study population. The baseline values were not continuous and did not fit the normal distribution curve. Kruskal Wallis test was performed for the between-group comparisons for the PAL task data. PALTEA, total error score was selected as the key measure for further analysis, a more universal metric due to its independence from the number of completed stages. Furthermore, to investigate whether the duration of LT-4 treatment influences the performance in associative learning, we divided Group 4, euthyroid – on LT-4 treatment, into 4a (having LT-4 for 1 or more but less than 5 years), and 4b (having LT-4 for a decade (10 years or more) for analysis of the key parameters of PAL task: PALTEA; PALFAMS. Subsequently, a linear regression analysis was performed, wherein the PALTEA score was taken as the dependent variable and adjusted covariables were: age, level of education and socioeconomic status. Next, general linear model analysis was performed for the key parameters of PAL task: PALTEA and PALFAMS, wherein independent variables were included: level of education and socioeconomic status.

## 3 RESULTS

**Fig. (1):**
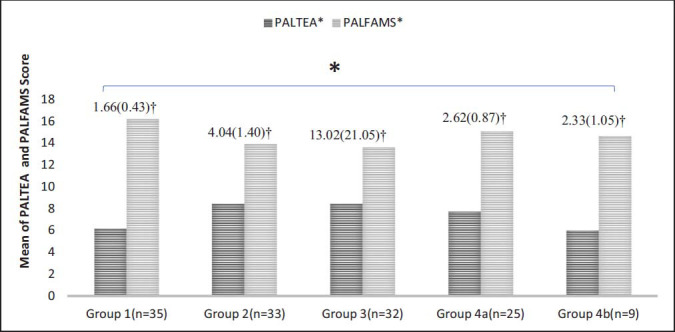
Comparison of total errors and first attempt memory scores to explore if duration of LT-4 treatment affects the performance in Paired association learning (PAL) task along with the reduction of TSH levels in Euthyroid patients. PALTEA- Paired association learning total errors adjusted, PALFAMS- Paired association learning first attempt memory score; * statistically significant differences between groups in the scores of PALTEA and PALFAMS (*p* = <0.05); † mean (standard deviation) of TSH (thyroid stimulating hormone) levels; Group 1- Controls, Group 2- Newly identified cases, Group 3-Patients with hypothyroidism symptoms (ongoing LT-4), Group 4a - Euthyroid (currently on LT-4 for 1 or less than 5 years), Group 4b - Euthyroid (currently on LT-4 for 10 or more years).

One thirty-four participants were included in the study, 99 patients diagnosed with SCH and 35 healthy controls. The demographic data of all participants and their clinical presentation are shown in Table **1**. 91.5% of study participants had an undergraduate or equivalent level of education and 55.4% of the participants had upper middle class socioeconomic status. The results between the groups of the PAL task variables were statistically significant, as mentioned in Table **2**. A significant trend was observed (Fig. **1**) in PALTEA and PALFAMS, 4b group made less errors as compared to 4a, indicating there was more enhancement in visual PAL and decrement of TSH levels after 10 years of LT-4 treatment in SCH population. Whereas Group 4a had a better PALFAMS score than group 4b, indicating enhanced new learning abilities in patients who were having medication for 1 or more than one year, see Fig. (**1**). Results of regression model (Table **3**) were statistically significant (F=4.89; *p* =0.003) after adjusting age (t= -0.69, *p* =0.48), level of education (t=-2.02, *p* =0.04) and socioeconomic status (t= -2.18, *p* =0.03). Also, the general linear model revealed that socioeconomic status and level of education were the predictor variables, may be influenced the results of PAL task data (PALTEA AND PALFAMS), as shown in Table **3**.

## 4 DISCUSSION

The specific deficit in associative or new learning in SCH has received scanty attention in literature despite its association with cognitive deficits. This is the first study to look into the impairment of visual paired associative learning or new learning in the SCH population.

Past research [4, 32] on SCH has strongly suggested that patients with SCH have impaired associative learning, untreated patients with hypothyroidism showed a distinct loss in memory retrieval [2]. In the clinic, it is observed that we can broadly classify patients with SCH into 3 categories, firstly patients who are reporting symptoms and have been newly diagnosed, secondly patients who have been diagnosed but are either non-compliant or poorly complaint regarding dosage of LT-4 hence, have poorly controlled TSH levels, thirdly patients who are compliant and are regular in follow up with the clinician’s office to regulate medication dosage to have TSH levels within normal limits. Hence, based on the clinical picture, it was decided to classify patients with SCH into 3 categories.

Findings of the present study show that PAL task was statistically significant (*p* = <0.05) impaired among patients’ groups as compared to controls.

PAL total error scores were significantly higher and first attempt memory score were lower in Groups 2, 3 and 4, which clearly indicates the impairment of memory retrieval in these patients as compared to control group 1, as shown in Table **2**. Among these patient groups; Group 3, patients on LT-4 treatment with elevated TSH (>4.0 mIU/L) and Group 2, newly diagnosed patients with TSH ≥ 2.5 mIU/L made more errors than Group 4, euthyroid patients on LT-4 treatment.

Thyroid hormones are known to alter the maturation of the cholinergic system, which is important in learning and memory [33]. As a result, as documented in animal model research [19], part of the effects of suboptimal levels of thyroid hormones on cognition could result in cholinergic system dysfunction. T4 therapy has been shown to improve the ability to learn memory tasks in adult rats, and these improvements were associated with increased cholinergic activity in the frontal cortex and hippocampus.

Patients in Group 2 and Group 3 performed poorly in PAL task in the present study. Three observational cross-sectional studies [8, 34, 35] have confirmed the presence of learning disorders and impaired memory in young patients with SCH. Additionally, a population-based study [36] has reported an association between AD and SCH. The data from existing literature [11, 12] suggest that when seeking to distinguish between healthy individuals and individuals with MCI or AD, the PALTEA score is a vitally important measure. They found higher levels of errors on PAL scores in patients as compared to healthy individuals. According to an ROC (receiver operating characteristics) analysis by another study [12], a cut-off score of 71 proved to be the most suitable distinguishing between individuals with amnestic MCI and AD and normal, age-matched adults. Along similar lines Hicks *et al*., 2021 reported a cut off score of 72. A very important finding in our study was that the PALTEA scores were substantially higher in Group 3 with a mean rank of 80.22, and in Group 2 with mean rank of 76.02, as shown in Table **2**, clearly indicating that visual paired associative learning is definitely impaired in SCH, as reaching levels seen in patients with MCI and AD.

The results of the generalised linear model in Table **3** reveal that both socioeconomic status and educational level exerted an influence on the outcomes in our current investigation. This aligns with prior research [37] that identified a selective impact of socioeconomic status on hippocampal-prefrontal reliant memory. People of low socioeconomic status have limited access to cognitively stimulating environments and cultural resources, which has a negative impact on their cognitive and executive functions. These functions deteriorate at different rates throughout life, with executive domains declining earliest and most rapidly. These functions also vary in the early stages of neurodegenerative diseases and respond differently to therapies, possibly influenced by socioeconomic status [38]. Recent findings [39] suggest that disadvantages in childhood socioeconomic status may negatively impact cognitive performance in old age.

Another interesting aspect of our study was that amongst the euthyroid patients, 25 out of 34 cases (with mean TSH 2.62±0.87 mIU/L), who were on LT-4 treatment for a duration of one or more but less than five years exhibited better first attempt memory scores, indicative of enhanced new learning potential, in comparison to those who had been on treatment for a decade, as shown in Fig. (**1**). In contrast, patients who had been using LT-4 for ten years or more with a mean of TSH 2.33 ± 1.05 mIU/L showed better results on PAL total error (adjusted) scores than those who had only been on the medication for a year or more than one year. A randomized clinical trial [40] conducted on patients with SCH (mean age 32.37 ± 11.35 years) concluded that there was an improvement in the associative learning component of Wechsler memory test after 3 months of LT-4 treatment along with a decrement of TSH levels, with a mean score of 2.00 ± 1.34 mIU/L. In another research [25], an improvement in memory recall and associative learning in the Digit symbol substitution test was observed when patients with an average age 31.67 ± 8.40 years achieved euthyroid state (TSH 3.4 ± 0.19 mIU/L) after 3 months of LT-4 treatment.

On the other hand, some research has indicated LT-4 has no effect on associative learning. A study by Parle and colleagues [26] documented that TSH levels in patients with SCH (mean age 73.5 ± 6.2 years) decreased after 6 months (median, (IQR) 4.0, (2.7-4.6 mIU/L) and 12 months (median, (IQR) 3.7 (2.8-4.9 mIU/L), but no improvement in associative learning when assessed with MEAMS (Middlesex Elderly Assessment of Mental state). In another study [15], there was no significant effect of LT-4 on associative learning in the Wechsler memory test after 12 months in patients with a mean age of 61.6 ± 11.5 years, but there was a reduction in TSH levels (with a mean of 1.52 ± 1.51 mIU/L).

The discrepancies among the studies regarding the effect of LT-4 treatment on associative learning in SCH population might be due to the age difference of the participants, where the improvement was found in the age group of 20-40 years but not found in the elderly population (age group 50-75 years).

Our results suggest that although SCH can result in learning deficits, treatment is critical and may resolve these deficits. Enhancement in associative learning or new learning performance due to the effect of LT-4 in Group 4 compared to Group 2 and Group 3 suggests the improvement in cognitive function in SCH treated subjects. It clearly indicates that the patients treating with hormonal replacement therapy to improve their cognitive state should be accepted. The present results could provide a SCH-specific cut-off point for TSH, which may help improve cognitive status of the patients. Also, the results can implicate clinical practice regarding the course of hypothyroidism (across different ranges and in euthyroid state) and the estimated long-term difficulties in cognition that patients might encounter.

## 5 LIMITATIONS OF THE PRESENT STUDY

First, subjects were not evaluated for dosage use of LT-4, which could also affect cognition. Second, the mean value of the HDRS scale was 5.09 in Group 2 (newly diagnosed patients with SCH), indicating that depression was not diagnosed in these patients, but the depressive symptoms have been pronounced in a high percentage (81.8%) in comparison to the other three groups of the present study, as shown in Table **1**. The potential impact of depressive symptoms on the performance of subjects in Group 2 should be considered. However, due to limitations, we were unable to analyse this effect on the PAL task performance.

Our findings encourage early and regular screening for visual associative learning or memory retrieval in patients with SCH, as well as TSH examination, in those who report fatigue, weight gain, depression, or memory problems. Results of our study also suggest in the presence of neuropsychological symptoms (fatigue, forgetfulness, insomnia, and depressive symptoms), treatment for SCH could start earlier at a threshold value of 2.5 mIU/L rather than taking a wait-and-watch approach.

Although this observational study contributes to our understanding of the specific impairment of paired learning in SCH in different TSH ranges and the improvement associated with LT-4 treatment, large randomized clinical trials with additional follow-up are still needed to confirm the results.

## CONCLUSION

In conclusion, our study clearly demonstrated that poor PAL task performance (specifically higher PAL total errors (PALTEA) scores) is exhibited in Group 2 and Group 3 patients with SCH, achieving levels demonstrated to be cut scores of patients diagnosed as AD [11, 12]. Thus, the results of our research encourage early examination of visual, associative learning or memory retrieval and the initiation of LT-4 treatment in patients with SCH, supporting the establishment of a more reasonable threshold of a TSH value of 2.5 mIU/L in patients exhibiting deficits in this test. Further research would help to characterize thyroid dysfunction in the preclinical stage of AD, which could enable a more sensitive and specific approach to early diagnosis and monitoring of disease progression.

## Figures and Tables

**Table 1:** Demographic data and clinical presentation of the study participants.

**Variable**	**Group 1** **(n=35)**	**Group 2** **(n=33)**	**Group 3** **(n=32)**	**Group 4** **(n=34)**
Age (years)	36.48 ± 9.51	37.24 ± 8.68	38.65 ± 10.81	39.70 ± 9.62
Gender	M=6; F=29	M=4; F=29	M= 1; F=31	M= 2; F= 32
Level of education	3.80 ± 0.79	3.60 ± 0.65	3.65 ± 1.03	3.52 ± 0.82
Socioeconomic status	20.65 ± 3.31	19.75 ± 3.77	20.59 ± 4.19	18.67 ± 4.92
MMSE	29.42 ± 0.73	29.18 ± 2.06	27.87 ± 2.02	27.55 ± 2.47
HDRS	1.94 ± 1.64	5.09 ± 3.71	4.43 ± 3.11	2.82 ± 2.19
TSH (mIU/L)	1.61 ± 0.48	4.04 ± 1.40	13.02 ± 21.05	2.54 ± 0.91
Range of TSH levels (mIU/L)*	1.60(0.70-2.30)	5.80(2.50-8.30)	104.91(4.45-109.36)	2.90(0.90-3.80)
T3(ng/mL)	1.57 ± 1.45	1.18 ± 0.58	2.59 ± 3.38	1.90 ± 2.35
T4(µg/dL)	7.68 ± 2.44	8.61 ± 1.49	9.01 ± 1.50	8.79 ± 1.74
Years of LT-4 treatment	-	-	3.30 ± 3.72	3.82 ± 3.55
**Neuropsychological and Other Symptoms**				
Fatigue	52.77%	72.72%	89.50%	57.14%
Weight Gain	16.5%	33.3%	69.9%	11.4%
Insomnia	22.2%	42.4%	59.3%	48.5%
Lack of memory	08.0%	78.78%	81.8%	42.8%
Depressive symptoms	13.8%	81.8%	71.8%	57.1%
Cold intolerance	0.5%	27.2%	32.5%	25.7%
Dry skin	11.1%	21.2%	36.3%	31.4%
Hearing loss	4.5%	18.1%	46.2%	11.4%

**Abbreviations:** MMSE-Mini Mental State Examination; HDRS- Hamilton Depression Rating Scale; TSH- Thyroid Stimulating Hormone; T3- Triiodothyronine; T4- thyroxine; M-Male; F-Female; Group 1 = Controls; Group 2 = Newly identified cases; Group 3= Patients with hypothyroidism symptoms (on going-LT-4); Group 4= Euthyroid (currently on LT-4); * highest value of TSH (minimum-maximum value of TSH).

**Table 2:** Comparison of paired associative learning (PAL) task parameters among all groups.

**Variables**	**Group 1 (n=35)** **Mean Rank**	**Group 2 (n=33)** **Mean Rank**	**Group 3 (n=32)** **Mean Rank**	**Group 4 (n=34)** **Mean Rank**	***p* Value**
PALTE	54.23	74.89	79.36	60.67	0.024*
PALTEA	55.00	76.02	80.22	60.13	0.019*
PALTA	60.60	75.91	79.09	55.53	0.028*
PALFAMS	86.93	58.30	53.05	70.03	0.002*
PALMETS	54.87	75.33	78.36	62.68	0.031*

**Abbreviations:** PAL- Paired associate learning; PALTE-PAL total errors; PALTEA-PAL Total Errors (adjusted); PALTA-PAL Total attempts; PALFAMS- PAL first attempt memory score; PALMETS-PAL mean errors to success; Group 1- Controls; Group 2- Newly identified cases; Group 3- Patients with hypothyroidism symptoms (ongoing LT-4); Group 4- Euthyroid (currently on LT-4); * statistically significant (*p* = ≤0.05).

**Table 3:** General linear model analysis (tests of between subjects effect), N=134.

**Independent Measure**	**Dependent Measure**	**df**	**MS**	**F**	***p***
Age	PALTEA	1	1.86	0.04	0.82
	PALFAMS	1	3.09	0.03	0.57
Level of Education	PALTEA	1	381.63	8.54	0.004*
	PALFAMS	1	71.39	7.70	0.006*
Socioeconomic status	PALTEA	1	381.39	10.36	0.002*
	PALFAMS	1	69.16	7.44	0.007*

**Abbreviations:** PALTEA-Paired association learning total errors (adjusted); PALFAMS: Paired association learning first attempt memory score; df- degree of freedom; MS- mean square; * statistically significant.

## Data Availability

The data and supportive information are available within the article.

## LIST OF ABBREVIATIONS

AD: Alzheimer’s Disease
CANTAB: Cambridge Neuropsychological Test Autom-ated Battery
LT-4: Levothyroxine
MCI: Mild Cognitive Impairment
PAL: Paired Associative Learning
SCH: Subclinical Hypothyroidism
TSH: Thyroid Stimulating Hormone

## STANDARDS OF REPORTING

STROBE guidelines were followed.
